# Comparing Distributions of Color Words: Pitfalls and Metric Choices

**DOI:** 10.1371/journal.pone.0089184

**Published:** 2014-02-25

**Authors:** Mikael Vejdemo-Johansson, Susanne Vejdemo, Carl-Henrik Ek

**Affiliations:** 1 Computer Vision and Active Perception Laboratory, KTH Royal Institute of Technology, Stockholm, Sweden; 2 Department of Linguistics, Stockholm University, Stockholm, Sweden; University of Leicester, United Kingdom

## Abstract

Computational methods have started playing a significant role in semantic analysis. One particularly accessible area for developing good computational methods for linguistic semantics is in color naming, where perceptual dissimilarity measures provide a geometric setting for the analyses. This setting has been studied first by Berlin & Kay in 1969, and then later on by a large data collection effort: the World Color Survey (WCS). From the WCS, a dataset on color naming by 2 616 speakers of 110 different languages is made available for further research. In the analysis of color naming from WCS, however, the choice of analysis method is an important factor of the analysis. We demonstrate concrete problems with the choice of metrics made in recent analyses of WCS data, and offer approaches for dealing with the problems we can identify. Picking a metric for the space of color naming distributions that ignores perceptual distances between colors assumes a decorrelated system, where strong spatial correlations in fact exist. We can demonstrate that the corresponding issues are significantly improved when using Earth Mover's Distance, or Quadratic 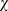-square Distance, and we can approximate these solutions with a kernel-based analysis method.

## Introduction

We study the effects of method choice for computational analyses of color semantics data sets.

Different languages have different numbers of color terms: English, for instances, has one main term for Blue: *blue*, while Italian divides Blue into two different shades: *azzurro* and *blu*. Throughout, we will use Small Caps for actual colors in the world and *italics* for color terms in specific languages. In order to study the behaviour of color names in different languages, Berlin & Kay [1] did a color term experiment with speakers of Arabic, Bulgarian, Catalan, Cantonese, Mandarin, English, Hebrew, Hungarian, Ibibio, Indonesian, Japanese, Korean, Pomo, Spanish, Swahili, Tagalog, Thai, Tzeltal, Urdu, and Vietnamese. Their study focused on the use of *basic color terms*: color names that should be monolexemic, not subordinate to another color term, not restricted to a narrow class of objects (*blonde*, in english, can only be used for hair, wood, and very few other objects) and psychologically salient for informants.

Remarkably, Berlin & Kay discovered that while languages have different numbers of basic color terms, the borders between them tend to run in approximately the same areas. Working without computational support, Berlin & Kay articulated a hierarchy of seven stages of color systems – establishing an order in which a color area detaches and forms its own color term across languages. Thus, they claim that for a language with only two color terms, the terms would divide the entire color space into a Dark-and-Cool and a Bright-and-Warm area. A language with three terms would divide the Bright-and-Warm, separating out a Red.

Subsequently, Kay, Berlin, Maffi and Merrifield [2] have collected a larger data set, the World Color Survey (WCS), to provide more detailed and reliable evidence on universal tendencies in color naming. The data set collects color names from 110 languages, gathering a total of 2 363 color terms from 2 616 speakers. The data was collected by displaying color sample chips. The collection contained 320 chips of maximally saturated color perceptually evenly distributed with regard to hue and lightness, as well as 10 chips along a grey scale.

This data set has subsequently been the subject of several computationally supported investigations into the properties of color naming schemes: Berlin & Kay [3] revise the color system hierarchy and Kay, Berlin, Maffi, Merrifield & Cook [2] find a collection of divisive principles that explain the partitions observed in the collected data. Lindsey & Brown [4] point out that previous studies focused on single color representatives. This entails computing an average representative of a distribution of responses. They argue that this practice occludes potentially important analysis data. Instead, they suggest that an analysis method that uses the entire distribution of response frequencies across the color samples will give a better understanding of the structures present in the dataset.

We agree with the basic point advanced by Lindsey & Brown. However, the methods they propose to deal with the problem still suffer from significant issues. In this article, we will detail these issues and propose a different analysis approach. We will also present a derivative dataset from the World Color Survey implementing our analysis approach. This will be freely available for future research through figshare[5].

## Methods

### Data representation

**Figure 1:**
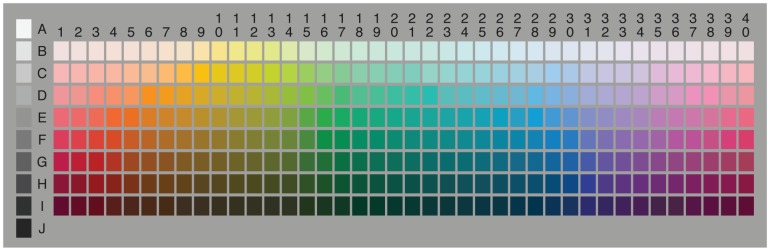
The Munsell color chart as used by the World Color Survey. All color distribution figures in this paper use this chart as a reference map; with darker grays for higher distribution intensity values and lighter grays for lower.

The World Color Survey [2] is a huge data-set that has been collected with the aim of understanding color naming in different languages. The data gathered during the survey can be represented by a two dimensional grid structure (see Figure 1) encoding the Munsell color system [6]. As said above, the Munsell color system has been chosen because the cells are perceptually evenly distributed. In order to quantify perceptual similarity between colors for the human visual system, the CIE standards association has developed a standard color representation scheme: the L*a*b* color space, where perceptual similarity is represented as spatial distances in the geometrical realization of the L*a*b* color space [7].

Every word in the dataset has been used by some speakers for a collection of cells in this grid structure. We will call this distribution of usage in the structure a *chart*. We illustrate charts in this paper by a gray-scale picture with darker gray for more frequent usages. One goal of the color naming research field is to investigate if and how words from different languages have similar charts. If this is so, a case can be made that there are *universals* underlying the language specific color naming systems.

The raw data of the responses in the World Color Survey has to be post-processed to enable effective statistical analysis. The method of post-processing is very important, as it determines the range of available statistical tools as well as their descriptive power.

We can note some approaches in use:

#### Centroid of responses

Regier and Kay [8] use a L*a*b* centroid method: in the CIE L*a*b* space the speaker chart for a particular word can be represented as a collection of points: the color space coordinates for the chips where this word was used by the speaker. By taking average values in each coordinate of the color space for these points, a speaker average color can be computed. Across all speakers of the language, a language average representative for the word can be computed by averaging the speaker average coordinates. The language average, finally, is projected out to a closest *centroid* Munsell cell. In this way the most representative grid cell for the given term in the given language is found.

#### Speaker response vector

The work by Lindsey and Brown [4] suggests instead to work with response distributions: Each speaker has responded with a term T to some subset of the 330 displayed color chips. The term is represented by a 330-dimensional vector with 0 when T was not used and 1 when T was used.

#### Language response vector

In the study by Jäger [9], all the speaker response vectors for a particular word in a language are summed up to form a language response vector.

While Kay and Regier have used the CIE L*a*b* space and its perceptual dissimilarities, the problem as pointed out by Lindsey and Brown, is that they used averages instead of the entire response distribution. The problem with Lindsey and Brown, and with Jäger, on the other hand is that the statistical methods they use ignore the underlying color space and the perceptual dissimilarities, which can be measured using for instance the CIE L*a*b* space.

We propose to combine the best of these studies: an awareness of perceptual distances in the CIE L*a*b* space with methods for handling distances between response distributions.

All the statistical methods in use depend on some way to compute a numeric *similarity* or *dissimilarity* between observations. Such a method produces a *metric space* structure on the data set. Mathematics provides us with a language and rich toolbox to understand and work with metric spaces. Therefore it is very beneficial to transform the observed representation into a metric space. This is a far from a trivial task and something that needs to be done with care as the metric needs to correctly reflect the underlying notion of structure in the data to guarantee that the statistics measures features of the data set instead of artefacts of the metric choice.

An easily accessible metric choice for response distributions would be the Euclidean distance function: 
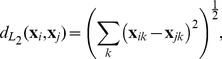
(1)where 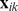 denotes chip 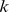 in chart 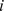. This approach was used as a means of analyzing the language response data in Jäger [9] for automating color naming analysis task through the means of Principal Component Analysis (PCA). In [4] the authors use the Pearson correlation coefficient as a similarity measure, computed as follows, 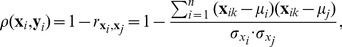
(2)where 
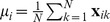
 and 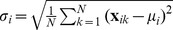 are the sample mean and standard deviation respectively.

However, both the Euclidean distance and the Pearson coefficient assumes that each of the chip responses, that is the 0–1 values for speaker response vectors or the response tallies for language response vectors are independent. Both measures are invariant to a permutation of the chips, which means that the responses can be re-ordered without influencing the dissimilarity measures. Clearly this is not the case as this would imply that the structure, the relative position of each chip in the chart, is irrelevant. Obviously this is not true as the chips have been structured such as to reflect the perceptual similarity between the colors they represent. This means that neither of these measures reflects that humans perceive Red as more similar to Pink than to Green. In practice this means that such distance measures will work well when comparing charts that are very similar, but will work less well once the responses are disjoint – no matter how perceptually similar or dissimilar these responses are.

In the remainder of this paper we will discuss the implications of different metrics used to study the World Color Survey data. We will highlight the assumptions that underlies specific metrics and show the implications they have on the results. Further we will suggest, in terms of application to the World Color Survey, a new set of metrics. We will argue for their relative benefits and show how using these metrics the interpretation of the data changes. As well as providing a basis for this paper, these representations are provided to other researchers as a foundation for future analysis of the data.

### Similarity measures

**Figure 2:**
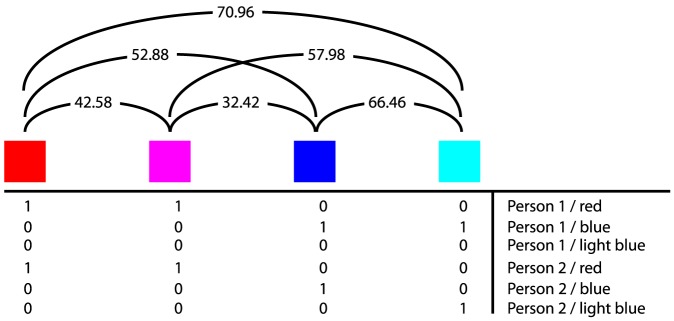
The two layers of data used here. At the top, we see a sample of four colors, as well as the CIE L*a*b* 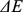 distances between all pairs among these colors. Below, we see a hypothetical sequence of color naming responses. Various papers by Kay and coauthors [2], [8], [17] pick out a best representative point in the space above and replace any distribution below with this single representative. Lindsey & Brown [4] point out that this approach, by ignoring most of the structural information contained in the concrete distributions in the data below loses significant information about color naming systems and patterns. Lindsey & Brown and Jäger [9] on their hand focus on the distributions of responses in the lower part of this figure, ignoring completely the perceptual distances in the color space above. In this paper, we argue that a better way is to work with methods that include both layers of the data: that work both with the perceptual distances above and the distributions below.

In this section of the paper we will focus on how to integrate the perceptual dissimilarities encoded in the CIE L*a*b* distance measure with the response data from the World Color Survey, in order to produce a similarity measure between different response charts. We illustrate the relationship between these layers in Figure 2.

We will focus on two different approaches to achieve this. In the first approach (Distribution Metrics), we leave the response vectors as is. We integrate the CIE L*a*b* space information in the algorithm that computes their similarity measures. In the second approach (De-correlation Mapping), we integrate the CIE L*a*b* space information into a transformation of the data itself, producing new vectors that can be further analyzed with classical statistical techniques.

### Distribution metrics

Each chart can be interpreted as a discrete distribution. There exists a significant body of work defining distance functions that can be applied on discrete distributions [10]. In order to avoid the negative characteristics of distance functions invariant under re-ordering (distance functions that assume that responses are independent) we wish for a distance measure for discrete distributions that respects the underlying distances of chips in the chart.

We will describe two candidates for a good distribution metric that takes the perceptual dissimilarity information into account: the Earth Mover's Distance and the Quadratic 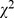 Distance.

Intuitively, if we consider the non-zeros values of the distributions as the mass of the distribution, a relevant distance measure should reflect the distance between the mass in two charts and not just their relative overlap. The Earth Mover's Distance measure [11] can be explained using a physical metaphor. If each distribution is thought of as a pile of dirt, the Earth Mover's Distance compares distributions by the amount of *work* that is needed to transform one distribution to the other. The work is defined by the amount of dirt times the distance it is moved. The minimal work needed can be computed using a linear program formulation. Even with industrial strength LP solvers [12], the amount of computation necessary to compute a full distance matrix becomes very large. We have done this for the *language response vectors* from the WCS data set, and are making the distance matrix available for further research. The computation, using CPLEX [12] for LP solutions took about 1 week on a single dedicated computer for the 2 300 language response vectors. Since the computation can be expected to scale quadratically, we consider it infeasible to use on the 23 982 speaker response vectors. This data can be used to approach the work by Jäger [9], since he uses the language response vectors for his analysis. The Earth Mover's Distance can not currently be used to approach the experiments by Lindsey & Brown [4], as they use the speaker response vectors.

A less computationally taxing method is the Quadratic 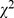 Distance. This was defined by Pele and Werman [13]. They propose to build on rescaled versions of the Euclidean metric – such as the 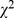 distance – that have significantly improved practical results in computer vision. These rescaling methods inspired a version that takes an underlying metric into account, defined through 
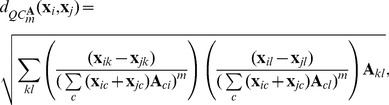
(3)where 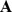 is a square symmetric matrix where element 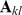 defines the similarity between chip 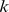 and 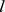 which can be acquired from a distance function between chips. Here, we write 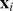 for the 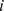
th chip vector and 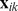 for the 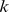
th entry in this vector. In [13] the authors apply the descriptor to a image retrieval task where the distance function was used to incorporate color similarity similar to what we are interested in here.

In our experiments we will be comparing the performance of these metrics on the World Color Survey dataset.

### De-correlation mapping

The distribution metrics introduced above are computationally intense and require specialized statistical techniques that can work with arbitrary metrics. The de-correlation mapping method will let researchers use more familiar tools and techniques for their analyses and is also much faster.

With the de-correlation mapping we seek to create a transformation of the response vectors themselves that encodes the CIE L*a*b* distances in the transformation. Perceptually close color stimuli produce responses that correlate closely. The transformation we introduce here controls for perceptual similarity, leaving only those correlations that are not directly explained by the perception of stimuli. Classical statistical techniques assume that there is no underlying systematic interdependence in the data, and this transformation makes the WCS data amenable to such techniques.

One method for constructing such transformations comes from the kernel family of machine learning techniques: for any bilinear form that behaves like an inner product (is positive semidefinite on all data samples), a linear transformation can be computed such that the bilinear form is the canonical inner product after transforming the vectors [14]. In particular, such a bilinear mapping can be constructed directly from a distance measure [15] by computing a Cholesky decomposition of the distance matrix: 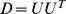. With such a factorization, the transformation 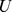 has the property that the inner product inducing the distance 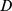 is the canonical inner product after applying 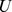.

The matrix of distances between the color chips used for elicitation produces such a bilinear form that models the expected correlation behaviour of distributions over these color chips. Thus, by computing a factorization of the color chip distance matrix, we produce a de-correlating mapping directly applicable on all kinds of charts.

This means that by decomposing a single 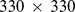 distance matrix using standard linear algebra techniques we can produce a transformation that enable classical statistical techniques while maintaining the influence of the color similarity measure itself.

### Cross dataset comparability

One added benefit of this added focus on the role of the underlying metric is that we can start comparing datasets that result from different experimental regimes. Both the Earth Mover's Distance and the Quadratic 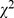 Distance allow us to easily and reliably compare distributions from different subsamplings of the color solid. The similarity matrix used to setup both the computations relies perceptual distances and can be formulated even if the subsamples for two different dataset are different.

The original study by Berlin and Kay [1] had 329 chips in their naming task, the World Color Survey has 330 and the EoSS study [16] uses 84 color chips. All these chips can be compared with the perceptual distance 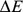 as defined by CIELAB, and thus the minimal work needed to transform from one of these distributions to another can be computed. Thus, this approach allows for data analysis between data collection instances and not only within a single experimental output.

## Results

On the one hand we will evaluate the family of available distribution metrics. On the other hand, we will demonstrate how to use the de-correlation mapping to revisit previous research on the data set.

### Evaluating metric choices: the example of Amuzgo

We have criticized several preceding research efforts for using inappropriate statistical techniques. We will now present some examples that demonstrate potential pitfalls with the choice of analysis methods. The following example illustrates the problem well.

**Figure 3:**
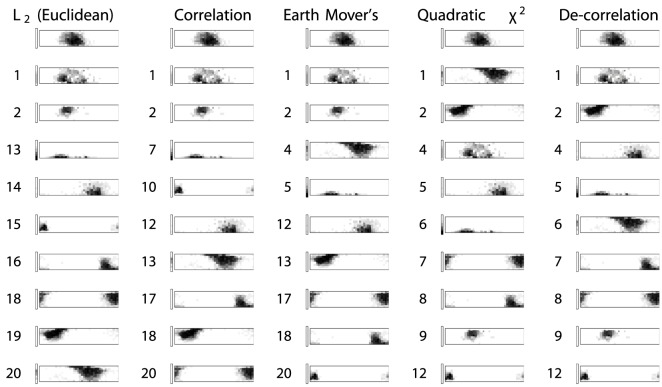
A selection of high-response rate Amuzgo color words ranked by different metrics by their distance from *cachii*. Ther permutation invariance of the metric used by Lindsey & Brown is visible in how the yellow/green immediate neighbour to *cachii* is placed 3rd most remote; while purple and disjoint red/brown and dark blue are placed far closer. The Amuzgo color words, as ranked by the 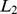 metric are on the far left. One might expect that neighboring color words should rank high in a metric that accurately compares distributions of color naming responses; however we can notice that the basic color words immediately neighboring *cachii* at the top left come *last* in the ranking.

The language Amuzgo has 21 color words with registered responses in the World Color Survey dataset. We pick as a reference point one of the commonly used words, *cachii*. In Figure 3, the color words of Amuzgo are ordered by their dissimilarity to *cachii* as computed by the different techniques we discuss in this paper.

A good distribution analysis method would rank perceptually similar color words closer than perceptually dissimilar words.

However, as can be seen in Figure 3, for the color words of Amuzgo, the opposite happens with the 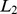 metric choice used by Jäger [9]. Out of the 21 used color words in the corpus, the immediately adjacent color terms in widespread use rank at places 13, 14, 19, and 20, while more distant words rank at 15, 16 and 18. The words that do rank close to *cachii* are precisely its partial synonyms, which is good, but also words with only very few mentions in the dataset, most of them far away from *cachii*.

For the correlation metric used by Lindsey & Brown [4], we see in the second column of Figure 3 that the immediately adjacent color terms referred above come in at ranks 7, 12, 13 and 18 while the more distant words discussed above come in at ranks 10, 17, 20. The positioning is somewhat improved from the 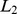 metric but still far from satisfactory.

As for the Earth Mover's Distance, we find the adjacent color terms at ranks 4, 5, 12, 13 while the more remote words come in at ranks 17, 18, 20. This is the overall order we would consider appropriate, and with a good separation between adjacent and remote distributions.

With the Quadratic 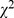 distance, the adjacent color terms show up at ranks 1, 2, 5, 6 while the more remote color words come in at ranks 7, 8, 12. This distance measure penalizes words with very small usages: the most remote words are those that have only very few responses at all.

Finally, after our de-correlation transformation we find the adjacent color words at ranks 2, 3, 5, 6, while the more remote color words are at ranks 7, 8, 12. A similar penalization for small usage seems to be in effect for this approach as well.

To summarize: the color words of Amuzgo illustrate how distribution metrics that are insensitive to perceptual distances end up ranking diametrically opposed regions of the color space closer to a sample color distribution than immediate neighbors. These ranking issues are especially important when the data is used for analysis methods with a global scope, such as the 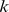-means clustering used by Lindsey & Brown, since an inaccurate ranking will group distributions together that do not belong in the same cluster. Both the 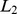 metric in use by Jäger [9] and the correlation metric used by Lindsey & Brown [4] suffer from these issues. The three approaches we discuss in this paper: using Earth Mover's Distance, using Quadratic 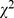, and using a de-correlation transformation all resolve the issues with the Amuzgo color words.

### Revisiting Lindsey and Brown: 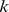-means clustering with de-correlation

Lindsey & Brown [4] use the speaker response vectors for a 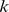-means clustering analysis to establish a hierarchy of naturally occurring clusters in the response data. As an example of how our suggested methods for analysis work in practice, we will revisit their investigation of the World Color Survey speaker responses and display a 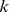-means cluster analysis that takes expected color response correlation into account before clustering. We will do these cluster analysis on two different data sets: first on the same data set that Lindsey & Brown analyzed, which excludes words with achromatic responses from the analysis based on a worry that minute changes in the achromatic axis might lead to larger changes in the rest of the analysis. We will then perform the cluster analysis on the full speaker response data, including words that have achromatic responses. Since we work with methods that integrate perceptual distances, we will demonstrate the beneficial effects of including these gray scale columns in the analysis.

We aggregate the WCS into speaker response vectors. For each speaker and word, there is a 0-1 vector picking out the color chips for which this was the word used by the speaker.

We construct a color chip similarity matrix 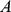 as 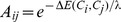
for color chips 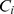 and 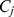, using the CIELAB-76 [7] definition for perceptual dissimilarity 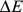. The parameter 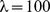 was chosen for giving a wide spread of numeric values for the resulting similarity values. We factor this matrix as 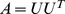 using Cholesky decomposition, and use the factor 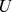 as a linear operator acting on the dataset.

After applying the operator 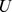, encoding color distances in the data, we use classical 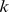-means clustering for continued analysis. The figures we display show sums of distributions belonging to a cluster. This way we can illustrate the cluster results using the original speaker response charts while performing the clustering in the transformed data set.

**Figure 4:**
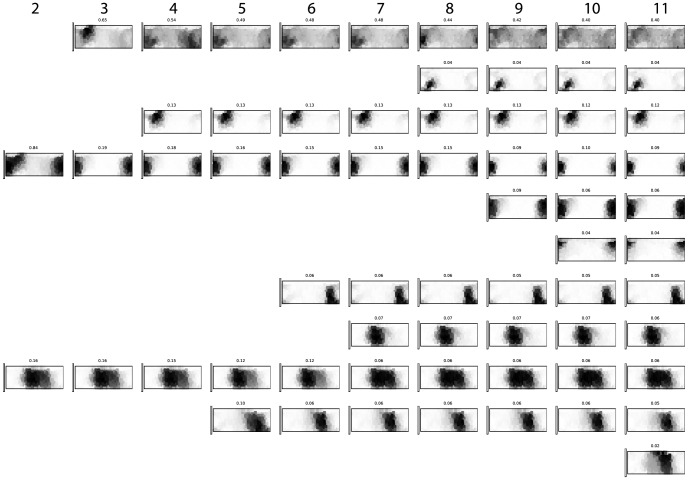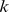-means clustering hierarchy on chromatic response vectors, after projecting by a factor of the exponential similarity matrix for color chips.

For the experiment performed by Lindsey & Brown, any response that includes any of the 10 achromatic chips are excluded from the analysis. This produces a dataset with 14 236 rows. We have performed a de-correlation transformation on this dataset, followed by a 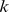-means clustering, in essence repeating the experiment of Lindsey & Brown using our analysis methods. We display our results in Figure 4.

**Figure 5:**
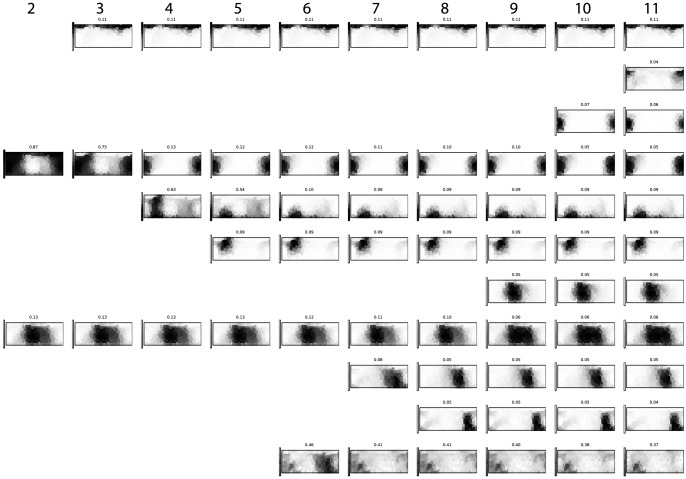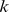-means clustering hierarchy on all response vectors, after projecting by a factor of the exponential similarity matrix for color chips.

For the full set of 21 992 speaker response vectors we show the results of 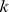-means clustering after de-correlation in Figure 5.

In each of the clustering experiments we have done, we repeated the 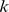-means clustering run 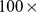 for each setting, using random initialization every time. Subsequently, we used a matching algorithm to pair up the cluster centroids. Each time all 100 iterations found the exact same collection of cluster centers.

Figure 4 and Figure 5 should be read from left to right with the data forced into one more cluster for each step to the right. Each plot is a copy of the Munsell chart, with darker grays for higher response frequencies.

As we can see in Figure 4, the kernel-based clustering of the chromatic color chips stays remarkably fixed: once a cluster center has been picked, it tends to remain approximately the same through higher and higher cluster numbers. Thus, we first see a split between warm and cold colors for the 2-means clustering. Thereafter, colors splinter off as we increase the number of cluster centers: for 3-means, there is a yellow-orange component that draws in responses from all over the spectrum in its cluster. At 4-means, a more distinct yellow-orange splits off from the catch-all cluster. Then, for 5-means, the cold splits into a predominantly green-focused grue and a blue-focused grue. At 6-means, the red remainder of the warm cluster spawns a a purple component. At 7-means, a distinctly green component splits out, leaving us with distinct clusters for green, blue and grue. At 8-means, a brown color splits out, mainly from the catch-all cluster, and at 9-means, the red component splits into a slightly darker part and a slightly lighter part. At 10-means, we see a pink component emerging, and at 11-means, a lighter blue shows up.

Where Lindsey & Brown [4] could observe color word clusters splitting and re-merging, the kernel-based approach avoids this puzzling behavior completely.

Lindsey & Brown state that

*We restricted our analysis to those color-naming patterns that were wholly contained within the 320 chromatic chips of the WCS chart, excluding all patterns that included any of the 10 achromatic chips (see Fig. 1a). We did so because an achromatic pattern, by definition, may differ from a chromatic pattern by only a single chip (black, white, or gray) drawn from a region of color space disjoint from those regions containing the chromatic chips. We were concerned that our clustering methods would be insensitive to these small but possibly (from a theoretical point of view) important differences in color naming.*

We expect that the insensitivity expected with their approach to clustering technique are taken care of by the new metric handling choices we present here – and thus, for comparison, we also computed 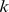-means clusters for the full collection of responses. We gathered 21 992 speaker response vectors from the World Color Survey dataset, and in Figure 5 we show the progression as it shows up for this larger dataset with the kernel based handling of the underlying metric.

Here, we can observe some concrete differences in the color progression. Again, we can observe that the found cluster centers are stable – once they show up, they tend to remind more or less unchanged through the range of cluster counts. Furthermore, we see that the splitting and re-merging behaviour reported by Lindsey & Brown is absent in this approach as well.

For two clusters, the WCS data set produces a split that does not entirely follow from Berlin & Kay's models: one cluster is a grue, and the other a kind of a catch-all cluster. Notably, both black and white have clustered with red. Instead of referring to these clusters as grue and red-black-white, we could call them cold and warm, confirming the finding of Kay, Berlin and Merrifield [17] that a cold/warm split is fundamental to color naming system evolution. However, Kay et al. first observe a bright/dark split, and only then a cold/warm split. In our analysis, the cold/warm split is the very first one observable. At 3-means, we recover a white cluster, and at 4-means, both grue and red have separated out from the white and catch-all categories. 5-means brings out a yellow-orange cluster, 6-means a purer brown-black separate from the catch-all cluster. At 7-means we see a blue separate out from the grue, and at 8-means a distinct purple. At 9-means, a green separates out as well, at 10-means a purer red, and at 11-means a pink.

To summarize, we have repeated the 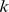-means clustering approach used by Lindsey & Brown [4] – both on the data set used by them and on the full speaker response vector data set from the World Color Survey. For both data sets, we have used the de-correlation transformation before clustering to encode expected color correlation into the data.

In the experiment performed with the same dataset as Lindsey & Brown we recover a different but similar hierarchy of color clusters as they did. Lindsey & Brown observed a curious behaviour of clusters splitting and merging as the cluster count increases: this problem disappears with the use of the de-correlation transformation.

In the extended experiment, using the entire speaker response vector set including achromatic color words, we can confirm and amend the finding of Kay, Berlin & Merrifield [17] that a splitting rule between cold and warm are important to the evolution of color naming systems. Lindsey & Brown are not in a position to say anything about the behaviour of dark and bright in relation to cold and warm since they have removed exactly these distributions from their analysis. Kay et al. postulate a few rules about this evolution, the primary being the splitting rule between dark and bright, and the secondary being the splitting rule between cold and warm. If 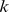-means clustering has a bearing on the evolution of color terms, our results indicate that the order of these two rules should be reversed.

## Conclusion

In this paper, we have presented concrete issues with commonly used approaches to the computational analysis of color naming data sets. As research into computational approaches to color semantics is done without a “cheat sheet” available to test results against, the impeccability of methods in use becomes all the more important to ensure reliability of any conclusions drawn from computational sources.

In particular, we have examined how the attention to distribution proposed by Lindsey & Brown can be combined with attention to the underlying perceptual metrics of color space.

We recommend against using color space agnostic methods – in particular PCA or clustering techniques directly on response distributions. Instead, we recommend techniques that integrate the structure of perceptual color dissimilarities into the analysis – such as is enabled by the distance datasets we publish or by a de-correlation transformation applied to the data.

These recommendations enable an approach to the analysis of color naming systems that can draw from all available data collection efforts – current or future – while maintaining comparability between different datasets.

We believe that the field of color linguistics is ripe for further fruitful inter-disciplinary collaboration efforts between field linguists, psycholinguistics and data sciences.
